# Interleukin-6 and uric acid among type 2 diabetes mellitus patients with coronary artery disease

**DOI:** 10.6026/973206300191134

**Published:** 2023-12-31

**Authors:** Gadigeppa H Chittaragi, Anand P Ambali, Rajesh Honnutagi, Veluri Ganesh

**Affiliations:** 1 Department of Medicine, Sri BM Patil Medical College Hospital and Research Centre, BLD & DU, Vijayapura, Karnataka, India; 2 Department of Biochemistry, ESIC Medical College and Hospital, Kalaburagi, Karnataka, India

**Keywords:** Coronary artery disease, type 2 diabetes mellitus, interleukin - 6

## Abstract

The type 2 diabetes mellitus is considering as metabolic disorder, the unfavourable long-term outcomes and closely associated with chronic inflammation. The present study investigated to explore the association of interleukin-6 and uric acid in patients with type 2 diabetes mellitus and coronary artery disease. Newly diagnosed CAD patients with T2DM (100) and T2DM and CAD patients after 5 years (100) underwent detailed anthropometric, demographic, biochemical and experimental characterization. The serum levels of interleukin 6 were measured by enzyme linked immuno-sorbent assay. The T2DM and CAD patients after 5 years had significant higher circulating levels of interleukin 6 and significant decreased levels of uric acid. The newly diagnosed CAD patients with T2DM had significant higher circulating levels of interleukin 6. Significant positive correlation was found between fat mass and IL-6, and negative correlation with uric acid and IL-6, (P<0.05). The both the groups of T2DM with CAD patients shown significantly elevated levels of interleukin 6, based on this findings interleukin 6 might be used as early predictable and prognostic marker for CAD in patients with T2DM.

## Background:

Coronary artery disease (CAD) is one of the most frequent cardiovascular illnesses that afflict people globally. It is brought on by atherosclerosis, an arteriosclerosis-related blood vessel condition [1-2]. The condition known as "hardening of the arteries," or arteriosclerosis, begins as soft fat deposits that over time solidify. More than one-third of deaths worldwide are caused by CAD, which is also on the rise in India. CAD is a major cause of death and disability in affluent nations [3-4]. The worldwide burden of illness study found that India had an age-standardized CAD death rate of 272 per 100,000 people, higher than the global average of 235 per 100,000 people. In the second half of the 20th century, the epidemic of coronary artery disease began to spread globally, especially in developing countries like India [5-6]. The biochemical markers were highly significant and had a strong relationship with coronary artery disease. High levels of IL-6 may also be a significant predictor of CAD.

Atherosclerosis, a disorder that affects the cardiovascular system, is a persistent inflammatory response of the body that encompasses stages of stability and instability. Large and medium arteries are where the illness first appears. CAD is caused by a build-up of fat and cholesterol in the arterial wall [7-8]. The development of plaque inner walls of the arteries causes atherosclerosis. This causes the arteries to harden and constrict. Blood flow to the heart decreases as plaque narrows the coronary arteries. As a result, the heart muscle's oxygen supply is reduced [9-10]. The circulating and local cytokine IL-6 plays a role in the development of CAD by influencing coagulant, endothelial, and metabolic processes. It has been discovered that IL-6 is related to the pathophysiology of ischemic cardiovascular events, such as unstable angina and ACS [11]. Angiotensin II-mediated vasoconstriction, oxygen radical release, and endothelial dysfunction can be caused by IL-6-induced up-regulation of angiotensin II type 1 receptor gene expression. IL-6 orchestrates the release of acute phase proteins and is essential for the acute inflammatory response [12]. By up-regulating acute-phase proteins, it plays a crucial part in the acute-phase response and the inflammatory cascade. Therefore, it is of interest to examine IL-6's ability to predict the existence of early coronary artery disease (CAD). Hence, the present study focuses on the role of serum IL-6 and uric acid levels and their association with CVD.

## Materials and Methods:

This cross -sectional study conducted in the department of general medicine and collaborated with biochemistry in -Sri BM Patil Medical College Hospital and Research Centre, Karnataka, India from 2021 to 2023. Two hundred patients diagnosed with CAD proven by angiogram were classified into two groups, Group - 1: Newly Diagnosed CAD in T2DM and Group - 2: T2DM and CAD after 5 years. All the study subjects were recruited after taken permission from Institutional Ethics Committee (IEC) of Sri BM Patil Medical College Hospital and Research Center and additionally we collected consent form from all the study subjects. Patients diagnosed with HTN, CAD without T2DM proven by angiogram, Patients diagnosed with HTN, CAD with T2DMproven by angiogram. All the subjects with age between 30 and 70 were included in this study. The subjects with history of thyroid disorders, liver disorders, kidney disorders, other types of heart disorders, immune related problems, and the subjects not willing to participate were excluded from this study.

## Collection of samples:

From all study participants we collected a venous blood sample of seven milliliters (mL) for the investigations. 2 mL of blood is transferred into an anticoagulant tube, 2 mL into an EDTA tube, and the remaining 3 mL into a plain tube after 8 to 12 hours of overnight fasting. After breakfast was consumed for two hours, another 2 mL of blood was taken for post prandial blood sugars. All samples that had been separated through centrifugation were put into aliquots and kept at -80°C until analysis was completed.

## Methods:

The fasting blood sugars (FBS) and post prandial blood sugars (PPBS) were analyzed by glucose oxidase peroxidase method and glycated haemoglobin (HbA1c) was measured by latex immuno turbidity metric method. Triglycerides was measured by enzymatic method and total cholesterol analyzed by cholesterol oxidase and peroxidase method. High density lipoprotein (HDL) is determined by selective inhibitory method and very low-density lipoproteins (VLDL), low density lipoproteins are calculated by friedewald's formula. Uric acid was analyzed by using uricase method and interleukins 6 was measured by enzyme linked immune assay (ELISA).

## Statistical analysis:

The Software Statistical Package for the Social Sciences (SPSS) Version 20.0 was utilized to conduct the statistical analysis. The data was presented as mean standard deviation. The three groups were compared using post hoc analysis, and the study subjects were compared using one-way analysis of variance (ANOVA). By using Pearson's correlation, the study's other parameters and BMI, uric acid and IL-6 were correlated. The statistical significance was set at P <0.05.

## Results:

The mean height, weight, and BMI of individuals in CAD group were significantly higher in T2DM with CAD after 5 years when compared to newly diagnosed CAD in T2DM, respectively P value is less than 0.001**. The mean values of FBS, PPBS, HbA1c, TGL, TC, VLDL, LDL significantly increased in T2DM with CAD after 5 years when compared to newly diagnosed CAD in T2DM (P<0.05). The HDL and uric acid concentrations were shown significantly decreased in T2DM with CAD after 5 years when compared to newly diagnosed CAD in T2DM, (P<0.05). the inflammatory cytokine interleukin - 6 was significantly elevated in T2DM with CAD after 5 years than the newly diagnosed CAD in T2DM patients, respectively P value is 0.001** (Table 1).

**Figure 1:**
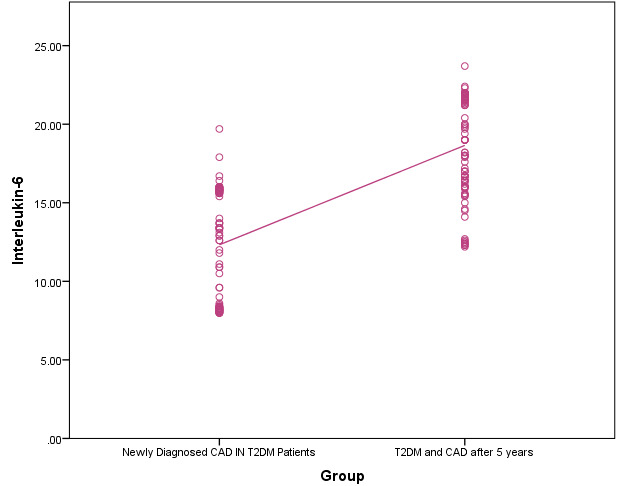
Comparison of distribution of interleukin 6 among two groups

**Figure 2:**
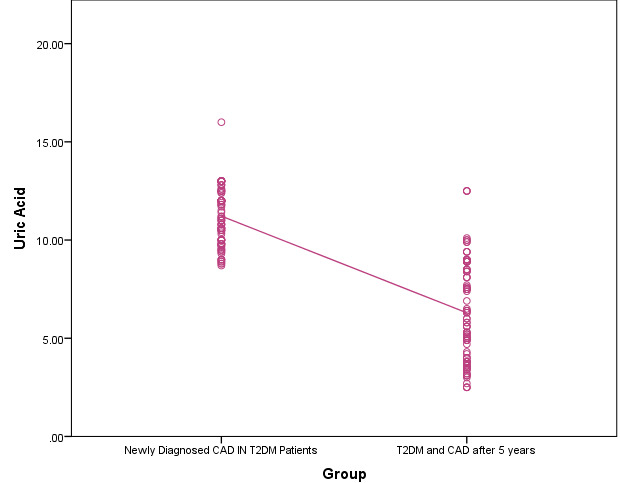
Comparison of distribution of uric acid among two groups

**Figure 3:**
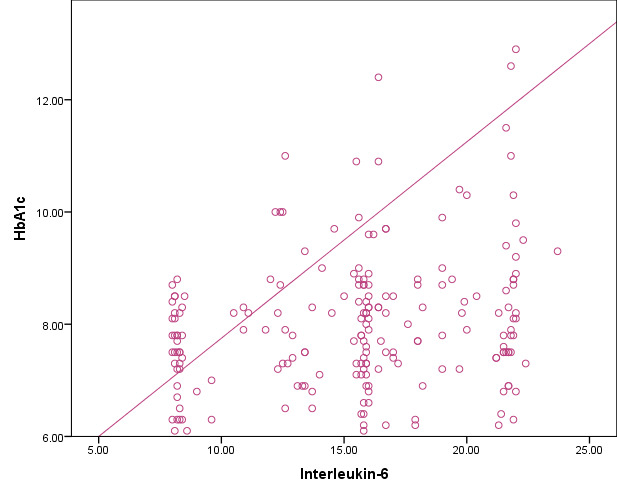
Comparison of HbA1c and interleukin 6 among two groups

Association between uric acid and IL-6 with other parameters in the study is tabulated in Table 2. There was a strong negative association between uric acid and BMI, FBS, PPBS, HbA1c, TC, TGL, VLDL, LDL, IL-6 and also found there was also a strong negative association between uric acid and HDL. The values were statistically significant (P<0.001). The association analysis of IL-6 (Figure 1) with the parameters in the study showed a strong positive association with all the parameters in the study except uric acid (Figure 2). The values were statistically significant (P<0.001). Figure 3 shows the scatter plots for the interleukin-6 and HbA1c concentrations among the study subjects. This revealed the interleukin - 6 was significantly positive association with HbA1c, the P value is <0.001.

## Discussion:

The elevated IL-6 levels were clear evidence of inflammation, which can be used to estimate the likelihood that a person will develop CAD. Studies show that, compared to CRP, IL-6 is a more sensitive and practical biomarker for the identification of cardiovascular disease. In other words, the higher the substance's plasma levels, the higher the mortality rate from heart disease. The severity of CAD, coronary events, mortality, and the onset of heart failure have all been associated to IL-6 levels [13].

The T2DM participants showed elevated levels of IL-6, which is directly related to the development of cardiovascular illnesses, according to numerous research. Additionally, we found that T2DM participants had considerably higher levels of IL-6, which was positively correlated with CAD. IL-6, a separate predictor of T2DM and associated cardiovascular events, has been connected to these occurrences [14-15]. Higher plasma IL-6 concentrations in patients with obesity and T2DM can reach 2-3 pg/mL1, which is primarily due to the adipocytes and macrophages located in adipose tissue. According to studies, persons with T2D have greater levels of IL-6 and CRP, which is likely because they have an abundance of adipose tissue [16]. In this type of tissue, macrophages secrete IL-6, which promotes fatty acid oxidation and, in turn, lipolysis. As a result, obese individuals-including those without T2DM-have higher IL-6 levels. Elevated levels of IL-6, which is well recognized to be a key stimulator of the production of many acute-phase proteins, were found to increase the risk of diabetes [17].

Serum IL-6 levels were found to be associated with increased BMI, high fasting insulin levels, and insulin resistance in type 2 diabetics. The rise in inflammatory cytokines is a key factor in T2DM-induced mitochondrial damage, oxidative stress, and beta cell death. Numerous researchers have discovered higher IL-6 levels in people with T2DM [18- 19]. Another earlier study discovered that T2DM patients had greater serum levels of IL-6 than did healthy people. The primary cardiovascular disease in T2DM or HTN patients is often coronary artery disease. People who had both HTN and T2DM were more likely to have cardiovascular events, according to past studies. Researchers have discovered higher levels of IL-6 in CAD patients compared to control participants. Large-scale investigations were undertaken by Liu et al. in China to investigate the combined impact of T2DM and HTN on the risk of cardiovascular events, and they discovered a high correlation between them [20]. The present study also observed, the IL-6 levels are significantly higher in patients with T2DM and CAD after 5 years when compared to newly diagnosed CAD patients with T2DM (P<0.05).

In this analysis, uric acid concentrations in group II shown a significantly decreased in patients T2DM and CAD after 5 years when compared to newly diagnosed CAD patients with T2DM (P<0.05). Along with that numerous epidemiologic studies have found associations between serum UA concentrations and a variety of cardiovascular conditions, including hypertension. Another recent study found that those with hypertension have greater uric acid levels than people with normal blood pressure. In hypertensive individuals, hyper uricemia is a unique predictor of early atherosclerosis.

Significantly increased weight, abnormal lipid profile results obesity. Now a day's obesity is common most significant risk factors for obesity related diseases. Many of T2DM patients are more prone to get CAD due to metabolic abnormalities, hyper glycemia, increased free radicals and cytokines. Based on the present study findings, the Interleukin 6, is one of the powerful predictors of coronary artery disease in patients with type 2 diabetes mellitus, since these values are significantly increased in later stages of T2DM and CAD when compared to newly diagnosed CAD patients.

## Conclusion:

Data shows that biochemical markers were highly significant and had a strong relationship with coronary artery disease. High levels of IL-6 may also be a significant predictor of CAD.

## Figures and Tables

**Table 1:** Demographic, anthropometric and biochemistry characteristics of study subjects

	**Newly Diagnosed CAD in T2DM**			**T2DM with CAD after 5 years**			
**Parameter**	**Mean**		**SD**	**Mean**		**SD**	**P- Values**
Age	48.2	±	6.16	53.34	±	6.11	0.001**
Height	3.12	±	0.45	2.94	±	0.39	0.001**
Weight	83.77	±	7.52	92.5	±	9.72	0.001**
BMI	27.3	±	4.16	31.89	±	4.57	0.001**
FBS	135.35	±	5.81	164.98	±	17.32	0.001**
PPBS	168.28	±	6	272.03	±	26.72	0.001**
HbA1c	7.57	±	0.82	8.48	±	1.38	0.001**
TGL	277.79	±	14.02	313.9	±	31.01	0.001**
TC	260.84	±	21.39	311.23	±	38.21	0.001**
HDL	32.8	±	5.13	30.07	±	3.25	0.001**
VLDL	55.59	±	2.83	62.77	±	6.21	0.001**
LDL	172.45	±	22.44	218.39	±	40.11	0.001**
Uric Acid	11.22	±	1.38	6.3	±	2.52	0.001**
Interleukin-6	12.33	±	3.53	18.65	±	3.17	0.001**

**Table 2:** Association between Uric Acid, Interleukin 6 and other parameters of the study

	**Uric Acid**		**Il-6**	
**Parameter**	**r**	**P**	**r**	**P**
BMI	-0.331	0.001**	0.307	0.001**
FBS	-614	0.001**	0.522	0.001**
PPBS	-0.711	0.001**	0.637	0.001**
HbA1c	-0.297	0.001**	0.238	0.001**
TGL	-0.568	0.001**	0.389	0.001**
TC	-0.48	0.001**	0.514	0.001**
HDL	0.242	0.001**	-0.234	0.001**
VLDL	-0.567	0.001**	0.386	0.001**
LDL	-0.423	0.001**	0.484	0.001**
URIC ACID	-	-	-0.326	0.001**
